# Supplementary material for: Facilitators and barriers to engagement with contact tracing during infectious disease outbreaks: A rapid review of the evidence
Source: PLoS One. 2020 Oct 29;15(10):e0241473. doi: 10.1371/journal.pone.0241473 (PMC7595276; doi:10.1371/journal.pone.0241473)
Supplement: S1 Table — (DOCX) [file pone.0241473.s001.docx]

**S1 Table: Search strategies**

Ovid MEDLINE(R) and Epub Ahead of Print, In-Process & Other Non-Indexed Citations and Daily 1946 to June 30, 2020

Date searched: 1^st^ July 2020

| # | Search terms | Hits |
| --- | --- | --- |
| 1 | Pandemics/ | 13506 |
| 2 | Coronavirus Infections/ | 13259 |
| 3 | Disease outbreaks/ | 79694 |
| 4 | "avian influenza".mp. | 10815 |
| 5 | Influenza A Virus, H1N1 Subtype/ or "bird flu".mp. or Influenza A Virus, H5N1 Subtype/ | 20652 |
| 6 | Coronavirus/ or coronavirus.mp. or covid 19.mp. or covid-19.mp. | 41596 |
| 7 | Ebola.mp. | 9108 |
| 8 | "equine influenza".mp. | 785 |
| 9 | H1N1.mp. | 21062 |
| 10 | H5N1.mp. | 7922 |
| 11 | MERs.mp. or Middle East Respiratory Syndrome Coronavirus/ | 4991 |
| 12 | "Middle East respiratory syndrome".mp. | 2409 |
| 13 | pandemic*.mp. | 42373 |
| 14 | Epidemics/ or epidemic*.mp. | 110203 |
| 15 | SARS Virus/ or SARs.mp. or Severe Acute Respiratory Syndrome/ | 19230 |
| 16 | "severe acute respiratory syndrome".mp. | 16107 |
| 17 | "swine flu".mp. | 958 |
| 18 | (contact* adj3 trac*).mp. | 6196 |
| 19 | exp Patient Isolation/ | 3895 |
| 20 | quarantine.mp. or exp Quarantine/ | 6128 |
| 21 | (self* adj3 isolat*).mp. | 872 |
| 22 | (survey* or (self* adj3 report*) or questionnaire*).mp. | 1311180 |
| 23 | (qualitative or interview* or focus group* or ethnograph* or fieldwork or field work or key informant).mp | 571548 |
| 24 | Or/1-17 | 256212 |
| 25 | Or/18-21 | 16600 |
| 26 | 22 or 23 | 1707675 |
| 27 | 24 and 25 and 26 | 625 |
| 28 | limit 27 to humans | 490 |
| 29 | limit 28 to english language | 446 |

APA PsycInfo 1806 to June Week 5 2020

Date searched: 1^st^ July 2020

| # | Search terms | Hits |
| --- | --- | --- |
| 1 | exp Pandemics/ | 677 |
| 2 | "avian influenza".mp. | 114 |
| 3 | "bird flu".mp. | 34 |
| 4 | coronavirus.mp. or covid 19.mp. or covid-19.mp. | 590 |
| 5 | Ebola.mp. | 420 |
| 6 | "equine influenza".mp. | 0 |
| 7 | H1N1.mp. | 498 |
| 8 | H5N1.mp. | 57 |
| 9 | MERs.mp. or "Middle East respiratory syndrome".mp. | 99 |
| 10 | pandemic*.mp. | 2283 |
| 11 | epidemic.mp. or exp Epidemics/ | 12790 |
| 12 | SARs.mp. or "severe acute respiratory syndrome".mp. | 537 |
| 13 | "swine flu".mp. | 93 |
| 14 | (contact* adj3 trac*).mp. | 199 |
| 15 | exp Social Isolation/ or quarantine.mp. | 7984 |
| 16 | (self* adj3 isolat*).mp. | 816 |
| 17 | (survey* or (self* adj3 report*) or questionnaire*).mp. | 779980 |
| 18 | (qualitative or interview* or focus group* or ethnograph* or fieldwork or field work or key informant).mp | 524665 |
| 19 | Or/1-13 | 15124 |
| 20 | Or/14-16 | 8897 |
| 21 | 17 or 18 | 1148803 |
| 22 | 19 and 20 and 21 | 49 |
| 23 | limit 22 to human | 42 |
| 24 | limit 23 to english language | 41 |

Embase 1980 to 2020 Week 26

Date searched: 1^st^ July 2020

| # | Search terms | Hits |
| --- | --- | --- |
| 1 | pandemic influenza/ or pandemic/ | 24259 |
| 2 | Coronavirinae/ | 2074 |
| 3 | "avian influenza".mp. or avian influenza/ | 14464 |
| 4 | "bird flu".mp. | 562 |
| 5 | coronavirus.mp. or covid 19.mp. or covid-19.mp. | 41621 |
| 6 | Ebola.mp. | 11813 |
| 7 | "equine influenza".mp. | 793 |
| 8 | H1N1.mp. or "Influenza A virus (H1N1)"/ | 29086 |
| 9 | H5N1.mp. or "Influenza A virus (H5N1)"/ | 10349 |
| 10 | Middle East respiratory syndrome coronavirus/ | 2280 |
| 11 | "Middle East respiratory syndrome".mp. or MERs.mp. | 6233 |
| 12 | pandemic*.mp. | 43529 |
| 13 | epidemic.mp. or epidemic/ | 162779 |
| 14 | severe acute respiratory syndrome/ | 8815 |
| 15 | "severe acute respiratory syndrome".mp. or SARs.mp. | 23535 |
| 16 | "swine flu".mp. or swine influenza/ | 1929 |
| 17 | exp Patient Isolation/ | 920 |
| 18 | quarantine.mp. or exp Quarantine/ | 5517 |
| 19 | (self* adj3 isolat*).mp. | 995 |
| 20 | (survey* or (self* adj3 report*) or questionnaire*).mp. | 2253942 |
| 21 | (qualitative or interview* or focus group* or ethnograph* or fieldwork or field work or key informant).mp | 728082 |
| 22 | Or/1-16 | 263629 |
| 23 | Or/17-19 | 7330 |
| 24 | 20 or 21 | 2753462 |
| 25 | 22 and 23 and 24 | 400 |
| 26 | limit 25 to humans | 354 |
| 27 | limit 26 to english language | 325 |

ProQuest (Coronavirus Research Database, Public Health Database, Social Science Database, Sociology Database and Internal Bibliography of the Social Science [IBSS])

Date searched: 1^st^ July

| # | Search terms | Hits |
| --- | --- | --- |
| 1 | noft(“avian influenza” or “bird flu” or Coronavirus or “covid 19” or covid-19 or Ebola or “equine influenza” or H1N1 or H5N1 or MERs or “Middle East respiratory syndrome” or pandemic* or epidemic* or SARs or “severe acute respiratory syndrome” or “swine flu”) |  |
| 2 | noft(contact* or trac* or isolat* or quarantine*) |  |
| 3 | noft(Survey* or self-report or questionnaire* or qualitative or interview* or focus group* or ethnograph* or fieldwork or field work or key informant) |  |
| 4 | 1 AND 2 AND 3 |  |
| 5 | Limit to Peer reviewed |  |
| 6 | Source type: Scholarly Journals; Document type: Article; Language: English | 1408 |

Also searched MedRxiv on: 15^th^ July

"(self-isolation OR quarantine) AND (survey OR qualitative)" (577 hits)

"(contact tracing) AND (survey OR qualitative)" (475 hits)
