# Supplementary material for: Facilitators and barriers to engagement with contact tracing during infectious disease outbreaks: A rapid review of the evidence
Source: PLoS One. 2020 Oct 29;15(10):e0241473. doi: 10.1371/journal.pone.0241473 (PMC7595276; doi:10.1371/journal.pone.0241473)
Supplement: S2 Table — (DOCX) [file pone.0241473.s002.docx]

**S2 Table: Excluded studies**

| **Reference** | **Reason for Exclusion** |
| --- | --- |
| Chung, S. C., Marlow, S., Tobias, N., Alogna, I., Alogna, A., & You, S. L. (2020). A rapid systematic review and case study on test, contact tracing, testing, and isolation policies for Covid-19 prevention and control. medRxiv. | Review (not appropriate to include in its entirety and checked for additional relevant primary studies) |
| Davalbhakta, S., Advani, S., Kumar, S., Agarwal, V., Bhoyar, S., Fedirko, E., ... & Agarwal, V. (2020). A systematic review of the smartphone applications available for coronavirus disease 2019 (COVID19) and their assessment using the mobile app rating scale (MARS). medRxiv. | Review (not appropriate to include in its entirety and checked for additional relevant primary studies) |
| Dimoulas, P., Green, K. A., Shigayeva, A., Aquino, M., McGeer, A., Scales, D. C., & Team, T. S. H. I. (2005). Patient contact recall after SARS exposure. Emerging infectious diseases, 11(4), 625. | No relevant outcomes |
| Gilmore, B., Ndejjo, R., Tchetchia, A., de Claro, V., Nyamupachitu-Mago, E., Lopes, C. A., & Bhattacharyya, S. (2020). Community engagement for COVID-19 prevention and control: A Rapid Evidence Synthesis. medRxiv. | Review (not appropriate to include in its entirety and checked for additional relevant primary studies) |
| Porten, K., Faensen, D., & Krause, G. (2006). SARS outbreak in Germany 2003: workload of local health departments and their compliance in quarantine measures—implications for outbreak modeling and surge capacity?. Journal of Public Health Management and Practice, 12(3), 242-247. | No relevant outcomes |
