# Supplementary material for: Facilitators and barriers to engagement with contact tracing during infectious disease outbreaks: A rapid review of the evidence
Source: PLoS One. 2020 Oct 29;15(10):e0241473. doi: 10.1371/journal.pone.0241473 (PMC7595276; doi:10.1371/journal.pone.0241473)
Supplement: S3 Table — (DOCX) [file pone.0241473.s003.docx]

**S3 Table: Quality appraisal of included studies**

Table (i): Quantitative surveys, assessed using the BMJ survey checklist

|  | Altman 2020 | Bachtiger 2020 | Filer 2020 | Helms 2020 | Jansen-Kosterink 2020 | Thomas 2020 |
| --- | --- | --- | --- | --- | --- | --- |
| Q1; Was a questionnaire the most appropriate method? | Can’t tell: Limited information | Yes | Can’t tell: Limited information | Can’t tell: Limited information | Yes | Yes |
| Q2: Have claims for validity been made and are they justified? | No | No | No | No | No | No |
| Q3: Have claims for the reliability been made and are they justified? | No | No | No | No | No | No |
| Q4: Are example questions provided? | Yes | Yes | Yes | No | Yes | No |
| Q5: Did the questions make sense, and could the participants in the sample understand them? Were ambiguous or overly complicated questions avoided | Yes | Yes | Yes | Can’t tell | Yes | Can’t tell |
| Q6: Are details given about the piloting undertake? | Yes | No | No | Yes | No | No |
| Q7: Was the questionnaire adequately piloted in terms of the method and means of administration, on people who were representative of the study population? | Can’t tell | Can’t tell | Can’t tell | Yes | Can’t tell | Can’t tell |
| Q8: Was the sampling frame for the definitive study sufficiently large and representative | Yes | Can’t tell, 4 days | Can’t tell, 8 days | Yes | Yes | Can’t tell, not reported |
| Q9: Was the method of distribution and administration reported | Yes | Yes | Yes | Yes | Yes | Yes |
| Q10: Were the response rates reported, including details of participants who were unsuitable for the research or refused to take part? | Yes | Yes | Yes | Yes | Yes | Yes |
| Q11: Have any potential response biases been discussed? | Yes | Yes | No | No | Yes | No |
| Q12: Was method of data analysis appropriate? (e.g. correct statistical tests for quantitative answers, qualitative analysis for open ended questions) | Yes | Yes | Yes | Yes | Yes | Can’t tell |
| Q13: Were all relevant data reported? | Yes | Yes | Yes | Can’t tell | Yes | Can’t tell |
| Q14: Are quantitative results definitive (significant), and are relevant non-significant results also reported? | No | Yes | No | No | Yes | No |
| Q15: Have qualitative results been adequately interpreted (e.g. using an explicit theoretical framework), and have any quotes been properly justified and contextualised? | NA | NA | NA | No | NA | NA |
| Q16: Have the researchers drawn an appropriate link between the data and their conclusions? | Yes | Can’t tell | Yes | Yes | Yes | Yes |
| Q17: Have the findings been placed within the wider body of knowledge in the field (e.g. via a comprehensive literature review), and are any recommendations justified? | Yes | No | No | Yes | Yes | No |

*NA: Not applicable*

Table (ii): Qualitative studies, assessed using the CASP tool

|  | Barker 2020 | Caleo 2018 | Greiner 2015 | Helms 2020 | Ilesanmi 2015 | Olu 2016 | Williams 2020 |
| --- | --- | --- | --- | --- | --- | --- | --- |
| Q1: Was there a clear statement of the aims of the research? | No, difficult to determine the aims of the study | Yes | Yes | Yes | Yes | Yes | Yes |
| Q2: Was a qualitative methodology appropriate? | Yes | Yes | Yes | Yes | Can’t tell: Limited details on methods | Yes | Yes |
| Q3: Was the research design appropriate to address the aims of the research? | Yes | Yes | Can’t tell: Limited details provided | Yes | No, only brief interviews and limited details on the questions asked | Can’t tell: Limited details on methodology | Yes |
| Q4: Was the recruitment strategy appropriate to the aims of the research? | Can’t tell: Methods poorly described | Can’t tell: Methods poorly described | Can’t tell: No details provided | Can’t tell: Methods poorly described | Can’t tell: No details provided | Can’t tell: Purposive sampling, risk of bias in selection of participants | Yes |
| Q5: Was the data collected in a way that addressed the research issue? | Can’t tell: Limited details on conduct of interviews or focus groups | Can’t tell: Limited details provided | Can’t tell: No details provided | Yes | Can’t tell: Limited details provided | Can’t tell: No details provided | Yes |
| Q6: Has the relationship between the researcher and participants been adequately considered? | Can’t tell: No details provided | Can’t tell: No details provided | Can’t tell: No details provided | Can’t tell: No details provided, simply states, “one male interviewer” | Can’t tell: No details provided | No: risk of bias as those conducting the interviews were involved with the organisations of contact tracers | Can’t tell: No details provided |
| Q7: Have ethical issues been taken into consideration? | Yes | Can’t tell: No details provided | Can’t tell: No details provided | Yes | Yes | Yes | Can’t tell: No details provided |
| Q8: Was the data analysis sufficiently rigorous? | Yes | Can’t tell: Limited details provided | Can’t tell: Limited details provided | Yes | Can’t tell: No information on analysis methods | Can’t tell: Limited information on analysis methods | Yes |
| Q9: Is there a clear statement of findings? | No: No clear summary provided | Yes | Yes | Yes | Yes | No: No clear summary | Yes |
| Q10: How valuable I the research? | Can’t tell: Limited details on how the research supports other findings | Yes | Can’t tell: Limited discussion provided | Yes | Yes | Yes | Yes |
