# Supplementary material for: Facilitators and barriers to engagement with contact tracing during infectious disease outbreaks: A rapid review of the evidence
Source: PLoS One. 2020 Oct 29;15(10):e0241473. doi: 10.1371/journal.pone.0241473 (PMC7595276; doi:10.1371/journal.pone.0241473)
Supplement: Attachment — Submitted filename: Response to reviewers.docx [file pone.0241473.s005.docx]

Response to reviewers

**Facilitators and barriers to engagement with contact tracing during infectious disease outbreaks: A rapid review of the evidence**

Dear reviewers,

Thank you very much for your time in reviewing our manuscript and for your very helpful comments and suggestions. We have responded to each of your comments below, and hope that we have addressed these to your satisfaction.

Many thanks,

Odette Megnin-Viggars

Reviewer #1: This study is a rapid review of contact tracing methods, to identify factors that are associated with greater engagement by patients. The research is interesting because it traces some facilitating elements and obstacles to contact tracing.

The manuscript is written in a clear and concise manner and provides agile information.

The authors refer to the PRISMA statement. However, these criteria apply to systematic reviews. The protocol they developed was not deposited on PROSPERO. Authors should say why they preferred to do a rapid review and which PRISMA criteria they did not follow. Thank you for your comment, this rapid systematic review satisfied all of the PRISMA checklist items, and this has now been clarified in the manuscript. We have also clarified in the manuscript that, unfortunately due to time restrictions, the protocol was not registered on PROSPERO.

Most of the retrieved studies concern Covid-19 and Ebola. The authors could tell us if there is a difference in the attitude of patients in these two diseases. For example, mistrust seems more frequent in Covid-19. Thank you for this interesting observation. I think this is true, but interpretation may be confounded by the fact that most of the COVID-19 studies included here focus on app-based contact tracing systems and the Ebola studies on manual contact tracing, so it is possible that it is mode of delivery differences rather than disease differences but I have added some additional text on page 22 of the manuscript to highlight these differences.

A few comments on the different geographical origin of the answers could also be interesting I agree this would be interesting, but unfortunately I do not think that the data allows such comparisons given that any geographical differences will be confounded by potential disease differences and mode of delivery differences. However, a prominent finding of this review was that there were a number of higher order conceptual themes that appeared to cut across studies. Previously we had conceptualized these as just cutting across mode of delivery, but I have now added some additional text to page 21 of the manuscript to highlight that these themes emerged irrespective of mode of delivery, *type of disease (COVID-19 and Ebola), and geographical region*.

Reviewer #2: This is a nice, concise and topical paper. Methods, recommendations and reporting are clear and relevant flow diagram and checklist for systematic review included. I support publication of this work. Thank you for your comments
